# Endoscopic ultrasound-guided hepaticogastrostomy using a 22G needle with a 0.018-inch ultra-stiff guidewire without tract dilation

**DOI:** 10.1055/a-2598-4134

**Published:** 2025-05-22

**Authors:** Takeshi Ogura, Jun Matsuno, Takafumi Kanadani, Ahmad F. Aboelezz, Hiroki Nishikawa

**Affiliations:** 1 Endoscopy Center, Osaka Medical and Pharmaceutical University Hospital, Osaka, Japan; 2 2nd Department of Internal Medicine, Osaka Medical and Pharmaceutical University, Osaka, Japan; 3 68781 Gastroenterology and Hepatology Unit, Department of Internal Medicine, Tanta University, Tanta, Egypt

Endoscopic ultrasound-guided hepaticogastrostomy (EUS-HGS) is indicated for the treatment of malignant biliary strictures in cases where attempts at biliary drainage under endoscopic retrograde cholangiopancreatography have failed. Recently, EUS-HGS is also increasingly being performed for benign biliary diseases, such as common bile duct or hepaticojejunostomy strictures 1 2 3 . However, since strictures cause greater narrowing of the intrahepatic bile duct than malignant biliary disease does, puncturing a stricture using a 19G needle is sometimes challenging. In such cases, using a 22G needle may enable successful puncture of the biliary tract – but there may still be a problem, because a 22G needle would require insertion of a 0.018-inch guidewire, the stiffness of which is less than that of the 0.025-inch guidewire. This could result in a longer procedure time, because the 0.018-inch guidewire needs to be exchanged for a 0.025-inch guidewire for the insertion of various devices.

Recently, a novel 0.018-inch ultra-stiff guidewire (J-Wire Premier NM, J-MIT, Shiga, Japan) has become available in Japan ( Fig. 1 ). This guidewire is made of a titanium, nickel, and cobalt alloy, and the sheath material is coated with polytetrafluoroethylene. The enhanced stiffness of this guidewire allows device insertion without the need to exchange the guidewire. Herein, we describe EUS-HGS using this guidewire.

**Fig. 1:**
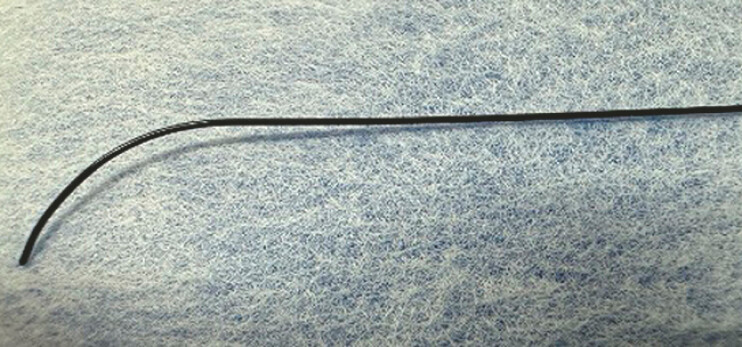
A novel 0.018-inch ultra-stiff guidewire made of a titanium, nickel, and cobalt alloy, with a sheath material coated with polytetrafluoroethylene.

A 59-year-old man, who had undergone pancreaticoduodenectomy because of pancreatic head cancer 3 years earlier, was admitted to our hospital due to complications of hepaticojejunostomy stricture and obstructive jaundice. EUS-HGS was attempted. Since the diameter of the intrahepatic bile duct was 1 mm on EUS imaging ( Fig. 2 ), a 22G needle was selected. Contrast medium was injected after successful bile duct puncture using the 22G needle ( Fig. 3 ), and the novel 0.018-inch guidewire was inserted and successfully deployed ( Fig. 4 ). Finally, a partially covered self-expandable metal stent delivery system was successfully inserted into the biliary tract without tract dilation, and was deployed from the intrahepatic bile duct to the stomach without any adverse events ( Fig. 5 ) ( Video 1 ).

**Fig. 2:**
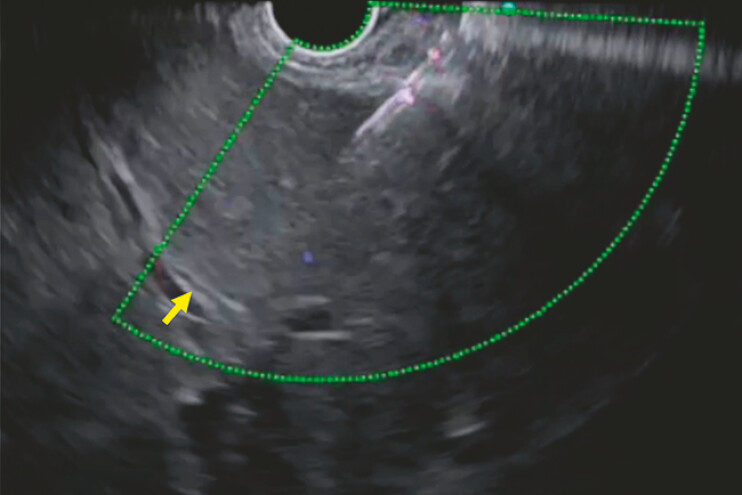
The diameter of the intrahepatic bile duct is 1 mm on EUS imaging.

**Fig. 3:**
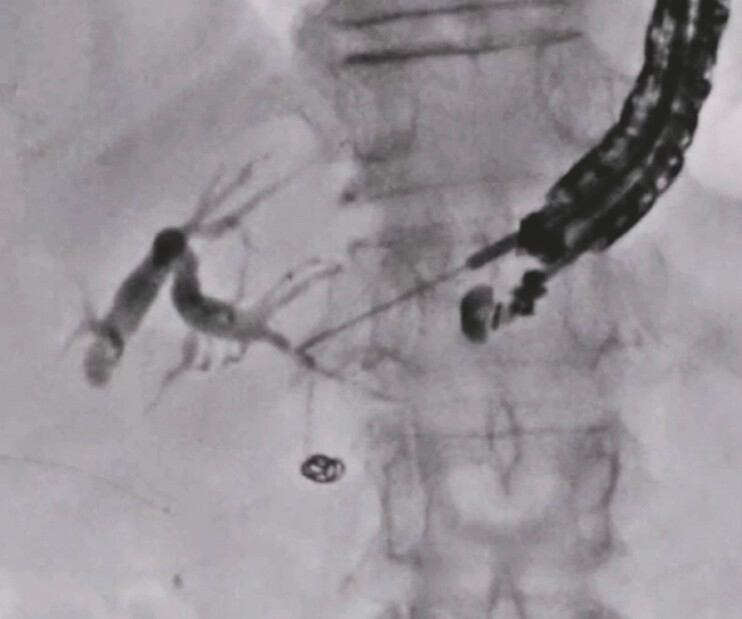
Contrast medium is injected after successful bile duct puncture using the 22G needle.

**Fig. 4:**
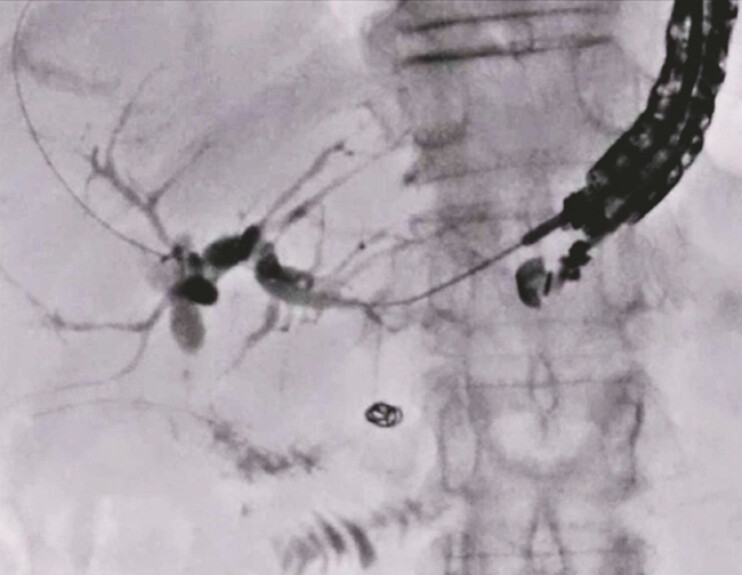
The novel 0.018-inch guidewire is inserted, followed by successful deployment.

**Fig. 5:**
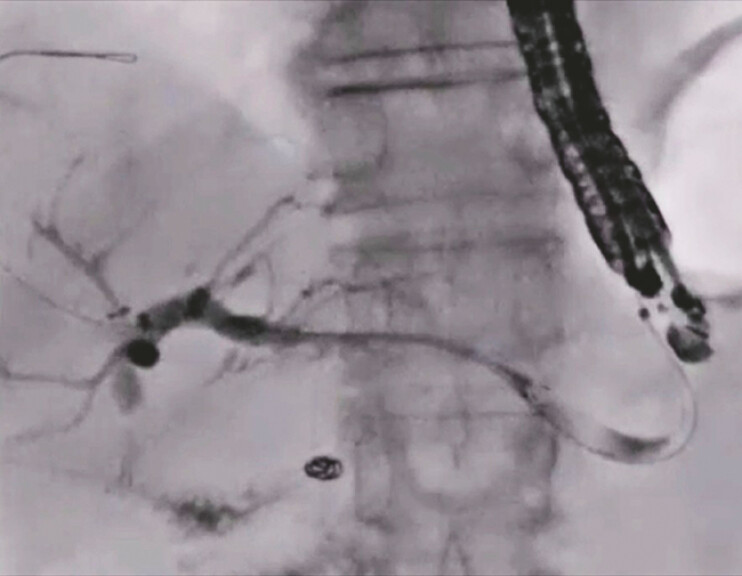
A partially covered self-expandable metal stent delivery system is successfully inserted into the biliary tract without tract dilation, and is deployed from the intrahepatic bile duct to the stomach.

The novel 0.018-inch guidewire is inserted and successfully deployed in an intrahepatic bile duct with an intraluminal diameter of 1 mm.Video 1

In conclusion, the new 0.018-inch ultra-stiff guidewire may be useful in cases in which a 22G needle is used for biliary puncture during EUS-HGS, as it may eliminate the need to change to a 0.025-inch guidewire.

Endoscopy_UCTN_Code_TTT_1AS_2AD

## Notes

Endoscopy E-Videos https://eref.thieme.de/e-videos
*E-Videos* is an open access online section of the journal *Endoscopy* , reporting on interesting cases and new techniques in gastroenterological endoscopy. All papers include a high-quality video and are published with a Creative Commons CC-BY license. Endoscopy E-Videos qualify for HINARI discounts and waivers and eligibility is automatically checked during the submission process. We grant 100% waivers to articles whose corresponding authors are based in Group A countries and 50% waivers to those who are based in Group B countries as classified by Research4Life (see: https://www.research4life.org/access/eligibility/ ). This section has its own submission website at https://mc.manuscriptcentral.com/e-videos .
